# Radiotherapy as an immunological booster in patients with metastatic melanoma or renal cell carcinoma treated with high-dose Interleukin-2: evaluation of biomarkers of immunologic and therapeutic response

**DOI:** 10.1186/s12967-014-0262-6

**Published:** 2014-09-23

**Authors:** Laura Ridolfi, Francesco de Rosa, Ruggero Ridolfi, Giorgia Gentili, Linda Valmorri, Emanuela Scarpi, Elisabetta Parisi, Antonino Romeo, Massimo Guidoboni

**Affiliations:** Immunotherapy Unit, Istituto Scientifico Romagnolo per lo Studio e la Cura dei Tumori (IRST) IRCCS, Meldola, FC Italy; Unit of Biostatistics and Clinical Trials, Istituto Scientifico Romagnolo per lo Studio e la Cura dei Tumori (IRST) IRCCS, Meldola, Italy; Radiotherapy Unit, Istituto Scientifico Romagnolo per lo Studio e la Cura dei Tumori (IRST) IRCCS, Meldola, Italy

**Keywords:** HD-IL-2, Melanoma, RCC, Radiotherapy

## Abstract

**Background:**

Tumor cells killed by radiation therapy (RT) are a potentially good source of antigens for dendritic cell (DC) uptake and presentation to T-cells. RT upregulates cell death receptors such as Fas/CD95 and MHC-I, induces the expression of co-stimulatory molecules on tumor cells, and promotes production of pro-inflammatory cytokines. High-dose interleukin-2 (HD-IL-2) bolus has been shown to obtain objective response rates ranging from 15% to 17% in patients with metastatic melanoma or renal cell carcinoma (RCC), with 6% to 8% of cases experiencing a durable complete response. However, HD-IL-2 is also associated with severe side-effects; if it is to remain a component of the curative treatment strategy in patients with metastatic melanoma or RCC, its therapeutic efficacy must be improved and patients who are most likely to benefit from treatment must be identified *a priori.* We designed a clinical study combining immunomodulating RT and HD-IL-2 to evaluate their clinical and immunological efficacy and to explore the predictive and prognostic value of 1) tumor-specific immune response and 2) serum levels of proangiogenic cytokines.

**Methods/design:**

The primary endpoint of this proof-of-principle phase II study is immune response. Secondary endpoints are the identification of biomarkers potentially predictive of response, toxicity, response rate and overall survival. Three daily doses of booster radiotherapy (XRT) at 6–12 Gy will be administered to at least one metastatic field on days −3 to −1 before the first and third cycle. Treatment with IL-2 (dose 18 MIU/m2/day by continuous IV infusion for 72 hours) will start on day +1 and will be repeated every 3 weeks for up to 4 cycles and then every 4 weeks for a further 2 cycles. Immune response against tumor antigens expressed by melanoma and/or RCC will be evaluated during treatment. Circulating immune effectors and regulators, *e.g.* cytotoxic T lymphocytes and regulatory T cells, as well as serum levels of proangiogenic/proinflammatory cytokines will also be quantified.

**Discussion:**

This study aims to evaluate the potential immunological synergism between HD-IL-2 and XRT, and to identify biomarkers that are predictive of response to IL-2 in order to spare potentially non responding patients from toxicity.

**Trial registration:**

EudraCT no. 2012-001786-32

ClinicalTrials.gov Identifier: NCT01884961

## Background

The concept that the immune system can distinguish the neoplastic from the normal self was proposed almost a century ago. Both cellular and humoral antitumor immune responses to tumor-associated antigens (TAAs) have been observed in many cancer patients. Tumors are genetically unstable, and the emergence of new genetic variants ensures that tumors survive in spite of the host’s immune system. Tumor development involves a prolonged series of checks and balances between the host, attempting to curtail tumor growth, and the tumor, benefitting from genetic changes, altering the microenvironment and avoiding immune elimination. Within this context, the “immunoediting hypothesis” suggests that the host’s immune system is capable of edit for the survival of tumors that become resistant to immune cells by eliminating cancer cells sensitive to immune attack [1-5].

Ionizing radiation therapy (RT) is known to kill cancer cells and other cells within the tumor stroma, including endothelial cells and intratumoral lymphocytes. The cell surface translocation of calreticulin or the extracellular release of high-mobility group protein B1 (HMGB1,a nonhistone nuclear protein), are among the molecular signals required to have an “immunogenic cell death”. Ionizing radiation may induce these changes and promote immunogenic cell death, favoring the development of an effective immune response [6-8]. Tumor cells killed by RT should, in theory, be a very good source of antigens for dendritic cell (DC) uptake and presentation of T cells [9,10]. The optimal activation of T cells by DC can only be achieved in the presence of inflammatory or “danger” signals. Although these danger signals can be generated by radiation exposure, their nature remains largely undefined [11]. Proinflammatory cytokines IL-1β and TNF-α can also be induced by radiation [12,13]. In addition, RT can upregulate cell death receptors such as Fas/CD95 and MHC-I and costimulate specific tumor cells, enhancing their tendency to either die or be recognized [14,15]. Finally, RT has complex effects on the tumor microenvironment: in particular, radiation-induced inflammatory signals, changes in extracellular matrix proteins, and the expression of adhesion molecules by endothelial cells of tumor vessels all facilitate the homing of both antigen-presenting and effector T cells to the tumor [16-18].

Demaria et al. recently showed in a murine model that irradiation of a grafted tumor generated an immune response against tumor cells and that this immune response inhibited the growth of tumor grafted outside the irradiation field. This anti-tumor effect was hypothesized to be the result of a specific immune response as it was not observed in immunodeficient mice (T-cell deficient, nude mice), was tumor-type specific (growth of a second graft of a different cell line was not affected by irradiation of the first graft), and synergized with administration of the FMS-like tyrosine kinase 3 (Flt-3) ligand. Indeed, the use of Flt-3 alone had no influence on the second graft, suggesting that the effect was not due to an unspecific boost of immunity induced by the Flt-3 ligand, but rather to the amplification of a tumor-specific response induced by the tumor irradiation. These data indicate that radiotherapy can prime the immune system against similar cells some distance away from the irradiation field (abscopal effect) and that, in the absence of an additional immune system stimulation, the radiation-induced immune response has no clinical effect [19-21]. However, optimal radiation regimens have yet to be defined. Formenti et al. compared different radiation regimens in combination with anti-CTLA-4 and found that a hypofractionated regimen (8 Gyx3) was more effective than a single dose of 20 Gy [8].

High-dose interleukin-2 (HD-IL-2) has been reported to induce an objective clinical disease regression in 15 to 17% of patients with metastatic melanoma and renal cell carcinoma, with 6% to 8% of cases experiencing a durable complete response of all metastases [22-24]. The first role attributed to IL-2 was its potent capacity to enhance in vitro T-cell proliferation and differentiation, and for this reason it was originally named T-cell growth factor. Following these initial descriptions of IL-2 function, a number of studies highlighted numerous contradictory functions of the cytokine. There is also evidence that IL-2 is an important factor in the generation of memory T-cells which undergo secondary expansion when they re-encounter an antigen. However, in contrast to these immune-enhancing functions, IL-2 also promotes activation-induced cell death (AICD) of T-cells and has anti-inflammatory properties [25].

Analogously to IFN-gamma, IL-2 limits IL-17 production and exerts its immunosuppressive function by stimulating the generation and homeostasis of regulatory T cells (Tregs) [26]. In fact, IL-2 is known to be a non-redundant factor in the in vivo homeostasis of Tregs which constitute a fundamental part of immunological self-tolerance and immune regulation [25]. IL-2 eradication of tumors is thought to be mediated by enhanced T-cell function and increased T-cell numbers. Lymphopoiesis is partially driven by lymphopenia and homeostatic proliferation. During homeostatic recovery, even in the absence of antigen stimulus, lymphocyte subpopulations shift, favoring antigen-experienced memory phenotypes and enhanced effector cell function. However, although the antitumor effect of HD-IL-2 is not completely understood, it appears to induce the release of proinflammatory cytokines into the blood and at the tumor site, enabling the ingress of inflammatory cells and promoting the cytolytic- and cytokine-producing activity of recruited lymphoid cells. This process drives the activation of monocytes/macrophages and other tumor-infiltrating immune cells, recruits immune cells from the periphery and triggers so-called *epitope spreading*, with novel determinants recognized in the context of the emergent immune response. HD-IL-2 suppresses CD4 + CD25hi-FoxP3+ T-regulatory cells, the prime contact-dependent suppressors of antigen-specific T cell response in metastatic melanoma patients, with a demonstrable clinical benefit.

Clinical evidence for an immune-mediated HD-IL-2 mechanism of action is based on tumor regression, the development of organ-specific autoimmunity in responders such as delayed-onset vitiligo, observed in some of our patients, and the onset of autoimmune thyroiditis. Thyroiditis was the first autoimmune disease reported to be induced by HD-IL-2 and which correlated with a favorable clinical outcome. As interleukin-2 paradoxically affects both tolerance and immunity in the absence of T-regulatory cells, HD-IL-2 increases effector CD8+ cytotoxic function which is responsible for both autoimmunity and tumor regression. Cancer immunotherapy with IL-2 has the potential to cure a subset of patients who need to be identified before treatment in order to minimize toxicity and improve therapeutic index [25,27,28].

Prospective clinical trials have reported a response rate of around 16% for melanoma patients treated with IL-2. Data suggest that higher rather than lower dose regimens are more likely to induce tumor regression in both melanoma and RCC. A retrospective analysis of 270 patients with metastatic melanoma treated with HD-IL-2 between 1985 and 1993 reported objective response rates of 16%, including a complete response rate of 6%. The median response duration was 6.5 months, and 60% of complete responders were progression-free after 5 years [29]. A major difference between the HD bolus IL-2 regimen and other chemotherapeutic melanoma therapies lies in the duration and quality of the responses obtained. Of the 24 patients receiving HD-IL-2 regimens at the National Cancer Institute who experienced a complete response (CR), 19 remained in complete remission for 46 to 137 months. In the past, several studies have focused on IL-2/chemotherapy combinations in metastatic melanoma. Although randomized trials have not been conducted to date, the HD-IL-2 bolus (600,000 to 720,000 U/kg intravenous (IV) every 8 hours for 15 doses) administered in the original NCI studies would seem to be superior to continuous IV infusion IL-2 [30]. Although response rates are comparable, they tend to be of shorter duration. In 7 published studies on metastatic RCC, objective responses (OR), complete responses (CR) and partial responses (PR) were seen in 15, 7, and 8% of patients, respectively. Furthermore, the median duration of response was 54 months for all responders, 20 months for PRs, and was not reached for CRs. The median survival was 16 months for all 255 patients [31]. However, HD-IL-2 administration is associated with numerous side-effects may thus have an impact on every organ system in the body. In fact, there is substantial variation in the toxicities experienced by patients receiving high-dose IL-2 which are thought to result primarily from capillary leak syndrome (CLS) and lymphoid infiltration, the latter observed histologically in many organs [32,33]. Sondel *et al.* studied continuous IL-2 infusion for 4–7 days at a dose of approximately 9 MIU/m2/day [34]. In addition, Dillman *et al.* demonstrated the efficacy of a higher dose continuous infusion IL-2 regimen (18 MIU/m2/day) over 5 days during which CLS proved to be the limiting toxicity [35]. The same group studied a 96-hour continuous infusion regimen for melanoma patients, reporting that it was better tolerated, with no decrease in response rate [36]. Continuous infusion may be more beneficial than bolus dosing in terms of inducing a higher degree of LAK cytotoxicity and higher rebound lymphocytosis. For these reasons, Quan *et al.* used the 72-hour high-dose continuous infusion schedule more frequently, reporting good tolerance and activity in both melanoma and renal cell carcinoma [37]. In an attempt to reduce the severity of CLS, Rosenberg’s group used concomitant corticosteroid infusion during IL-2 therapy, observing that the cytotoxic function of CD8 + T lymphocytes was not affected and that side-effects were reduced [38]. It is clear that, if HD-IL-2 is to remain a component of a curative treatment strategy in patients with metastatic melanoma or RCC, its therapeutic index will have to be improved.

Two active areas of investigation within the field of immunotherapy focus on the elimination of immune suppression/regulation and the improvement of patient selection.

Based on an understanding of the mechanisms of action of IL-2, biomarkers of response to this cytokine might allow us to identify a subset of patients who could potentially benefit from treatment. To date, the majority of putative predictors of IL-2 response have been post-treatment variables, such as the height of rebound lymphocytosis, treatment-induced thrombocytopenia, the development of autoimmune thyroiditis and vitiligo, and the decrease in the absolute number and frequency of peripheral Tregs.

In renal cell carcinoma, the level of carbonic anhydrase IX (CAIX) in primary tumors has been found to be a potentially useful pretreatment predictor. In both melanoma and RCC, serum vascular endothelium growth factor (VEGF) and fibronectin have also been identified as independent predictors of non response, and high levels of these proteins have been correlated with a lack of clinical response and decreased overall survival. Other analyses of pretreatment factors have correlated IL-2 with the Cw7 phenotype or low serum levels of IL-6 and C-reactive protein, but none has proven to be sufficiently predictive of clinical benefit to be useful in patient selection for IL-2 therapy [39-44].

## Methods/design

### Objectives

This is a proof-of-principle phase II study to assess 1) immune response and 2) potential biomarkers predictive of response to treatment with HD-IL-2.

### Primary objective

The primary objective of this study is to determine the immunological efficacy of the combined RT/HD-IL-2 treatment (assessed by IFN-g ELISPOT analysis) in terms of its ability to enhance the proportion of a selected panel of circulating immune effectors specific for tumor antigens known to be expressed in RCC and/or melanoma.

### Secondary objectives

1. to prospectively determine the predictive value of pretreatment biomarkers in identifying patients who could potentially benefit from combined RT/HD-IL-2 treatment, *i.e.* serum levels of VEGF-A, fibronectin, proinflammatory cytokines (IL-1beta, IL-6, IL-8, IL-10, IL-12, alphaTNF), tumor tissue expression of CAIX for RCC and presence of N-Ras mutation for melanoma.
2. Toxicity
3. Response rate
4. Overall survival

### Trial design

#### Summary of trial design

Single center, open-label proof-of-principle phase II trial to assess immune response and predictive biomarkers of response.

Three daily doses of booster radiotherapy (XRT) at 6–12 Gy to 1–5 metastatic fields will be administered on days −4 -3 -2 or −3 -2 -1 before the first and the third cycle of IL-2. IL-2 will be administered on day +1 of each cycle.

Treatment with IL-2 (dose 18 MIU/m2/day in 500 cc by continuous IV infusion for 72 hours) will start on day +1 and will be administered every 3 weeks for up to 4 cycles, then every 3–4 weeks for a further 2 cycles.

- Total duration of the trial: 36 months:
- Enrollment period: 20 months
- Treatment: maximum of 6 cycles (5 months) per patient
- Follow-up every three months
- Step 1: 7 patients will be enrolled; on the basis of a minimax two-stage Simon design, a 40% immune response will preclude further study, whereas a 70% response rate will indicate that further study is warranted. Using alfa and beta errors of 0.10, if an immune response is observed in at least 3 of the 7 patients enrolled during the first stage, the study will go on with:
- Step 2: recruitment of a further 12 patients.

The treatments will be considered active if an immune response is observed in 11 out of 19 patients treated. The analysis will be performed on an intention-to-treat population, *i.e.* all patients receiving at least one cycle of therapy.

### Primary and secondary endpoints/outcome measures

*The primary objective of this study will be:*

- **immunological efficacy**, assessed by quantification of circulating immune effectors specific for a selected panel of tumor antigens. In particular, this panel will include tumor antigens known to be highly expressed in over 80% of patients with melanoma or RCC in and whose expression must be confirmed in tumor biopsies taken before and after at least 2 cycles.

*The secondary objectives will be*

- **assessment of the predictive value of pretreatment serum biomarkers** in identifying patients who will probably benefit from high-dose IL-2 based therapy. In particular, pretreatment concentrations of VEGF, fibronectin and proinflammatory cytokines (IL-1beta, IL-6, IL-8, IL-10, IL-12, alphaTNF) will be evaluated by SearchLight multiplex array analysis. Expression of CAIX in pretreatment biopsies from RCC patients will be evaluated by immunohistochemistry. In melanoma patients, tumor tissue biopsied before the start of treatment will be evaluated for the presence of N-Ras mutations.
- **toxicity, response rate and overall survival.**

Patients will be evaluated every 8 weeks by computed tomography imaging to determine the clinical response, and every 3 months after completion of treatment until death. The time of disease progression and the initiation of alternative therapies will also be recorded.

### Study population

Patients with metastatic melanoma or renal cell carcinoma with measurable disease, life expectancy of at least 3 months and with tissue sample availability.

### Inclusion criteria

1. Patients must have histologically or cytologically confirmed non resectable stage III or IV advanced melanoma or renal cell carcinoma (RCC).
2. Patients must have a minimum of two lesions, one of which must be measurable (*i.e.* a lesion that can be accurately measured in at least one dimension with longest diameter >20 mm using conventional techniques or >10 mm using spiral CT scan).
3. At least one tumor lesion accessible for bioptic sampling.
4. Prior lines (maximum 4) of chemotherapy, immunotherapy or biological therapy (*e.g.* inhibitors of B-Raf or c-Kit, ipilimumab, etc.) for advanced disease are allowed (patients must have finished prior treatments at least 4 weeks before the first IL-2 dose).
5. Male or female, aged >18 years.
6. Life expectancy > 3 months.
7. ECOG performance status <1.
8. Patients must have normal organ and marrow function:
  ECG and echocardiogram within normal institutional limits.
    Pulmonary function tests within normal institutional limits (only to be performed in patients with lung metastases or history of impaired lung function).
9. No contraindication for the use of vasopressor agents.
10. Female participants of child bearing potential and male participants whose partner is of child bearing potential must be willing to ensure that they or their partner use effective contraception during the study and for 3 months thereafter.
11. Participant is willing and able to give informed consent for participation in the study.

### Exclusion criteria

The participant may not enter the study if ANY of the following apply:

1. Patient with stage I or II melanoma or RCC.
2. Patients who have had chemotherapy or radiotherapy or immunotherapy within 4 weeks (6 weeks for nitrosoureas or mitomycin C) prior to entering the study or those who have not recovered from adverse events due to agents administered more than 4 weeks earlier.
3. Participation in another clinical trial with any investigational agents within 30 days prior to study screening.
4. Patients with known brain metastases should be excluded from this clinical trial because of their poor prognosis and because they often develop progressive neurologic dysfunction that would confound the evaluation of neurologic and other adverse events.
5. History of allergic reactions attributed to compounds of similar chemical or biologic composition to IL-2 or other agents used in the study.
6. Any autoimmune disease which could be exacerbated by IL-2.
7. A medical illness requiring chronic treatments with corticosteroids or other immunosuppressive agents.
8. A history of significant cardiovascular disease, including myocardial infarction, congestive heart failure, primary cardiac arrhythmias, angina pectoris or cerebrovascular accident.
9. Uncontrolled intercurrent illness including, but not limited to, ongoing or active infection, symptomatic congestive heart failure, unstable angina pectoris, cardiac arrhythmia, or psychiatric illness/social situations that would limit compliance with study requirements.
10. Other known malignant neoplastic diseases in the patient’s medical history with a disease-free interval of less than 5 years (except for previously treated basal cell carcinoma and *in situ* carcinoma of the uterine cervix).
11. HIV-positivity, whether or not symptomatic.

### Study treatment

#### Radiotherapy

Radiotherapy will be administered in three daily doses of 6–12 Gy (depending on the volume and anatomic location of irradiated lesion) to 1–5 non-index metastatic fields. The reference isodose will be 60-70%/isodose, with an increased dose distribution inside the tumor. Radiotherapy techniques permitting a rapid decrease of the dose distribution outside the target (IMRT-IMAT) will be used. Pre-treatment XRT will be scheduled to finish on day −2 or −1. Radiotherapy will be performed twice, before the first and third IL-2 cycles.

#### High-dose IL-2

The treatment is based on high-dose IL-2: 18 MIU/m2/day in 500 cc administered by continuous IV infusion over 72 hours. The administration of IL-2 will take place on an inpatient basis. The first day of IL-2 therapy is considered as Day +1. Treatment with IL-2 will be administered every 3 weeks for up to 4 cycles, then every 4 weeks for a further 2 cycles.

#### Concomitant medications

During the 3rd day of IL-2 infusion, the following will be administered:

- 3 bolus/day of desametazone 4 mg;
- Ondansetron 8 mg ×2/day;
- Paracetamol 500 mg ×3/day;
- Adequate i.v. hydration.

### Dose delays/modifications

NCI Common Toxicity Criteria version 4.0 (available at: http://www.eortc.be/services/doc/ctc/CTCAE_4.03_2010-06-14_QuickReference_5x7.pdf) will be used. No dose reductions of IL-2 are allowed. Dose delays within the same cycle: IL-2 doses will be delayed on the basis of the symptomatic recovery from the previous dose. A delay longer than 24 hours will result in discontinuation of that cycle of IL-2.

### Study treatment modifications

Guidelines for delay or discontinuation of IL-2 are given in Tables 1 and Table 2. The presence of relative criteria implies that a patient is nearing the completion of a cycle of therapy and that with appropriate corrective measures or with a time delay to allow for recovery, it may be safe to administer another IL-2 dose. Several relative criteria that are not easily reversible or correctable are usually an indication that treatment should be discontinued. The presence of an absolute criterion that is not easily reversible is also generally considered an indication to interrupt IL-2.

**Table 1:** **Guidelines for delay or discontinuation of IL-2: relative and absolute criteria**

**System**	**Relative criteria**	**Absolute criteria**
***Cardiac***	- Sinus tachycardia (120–130 beats per minutes);	- Sinus tachycardia (>130 beats per minutes persists after correcting hypotension, fever and stopping dopamine);
		- Atrial fibrillation;
		- Supraventricular tachycardia;
		- Ventricular arrhythmia;
		- Elevated creatine kinase isoenzymes or troponin;
		- Electrocardiogram changes of ischemia;
***Dermatologic***	---	- Moist desquamation;
***Gastrointestinal***	- Diarrhea, up to 6 episodes/day	- Diarrhea, > 6 episodes/day
	- Ileus/abdominal distension;	- Severe abdominal distention affecting breathing;
	- Bilirubin >7 mg/dl;	- Severe abdominal pain, unrelenting;
***Hemodynamic***	- Maximum neosynephrine 1–1.5 mcg/kg/min;	- Maximum neosynephrine 1.5-2 mcg/kg/min;
	- Maximum neosynephrine >0.5 mcg/kg/min;	- Maximum neosynephrine >0.8 mcg/kg/min;
***Hemorrhagic***	- Guiac + sputum, emesis, stool;	- Frank blood sputum, emesis, stool;
	- Platelets 30,000-50,000/mm^3^;	- Platelets <30,000/mm^3^;
***Infections***	---	- Strong clinical suspicion or documented;
***Muscoloskeletal***	- Weight gain >15%;	
	- Extremity tightness;	- Extremity paresthesias;
***Neurologic***	- Vivid dreams;	- Hallucination;
	- Emotional lability;	- Persistent crying;
		- Mental status changes not reversible in 2 hours;
		- Inability to subtract 7 s or spell “world” backwards
		disorientation;
***Pulmonary***	- Resting shortness of breath;	- >4 L O2 nasal cannula for saturation >95% or 40% O2 mask for saturation >95%;
	- 3-4 L O2 nasal cannula for saturation >95%;	- Moist rates involving more than half of both lung fields;
	- Moist rates involving more than half of both lung fields;	- Endotracheal intubation;
		- Pleural effusion requiring tap or chest tube;
***Renal***	- Urine 80–160 ml/shift;	- Urine <80 ml/shift;
	- Urine 10–20 ml/h;	- Urine <10 ml/h;
	- Creatinine 2.5-2.9 mg/dl.	- Creatinine >3 mg/dl.

**Table 2:** **Guidelines for delay or discontinuation of IL-2 based on the number of assessed relative or absolute criteria**

**Observation category**	**Action**
***Any relative criteria***	- Initiate corrective measure +/− delayed IL-2;
***Three criteria***	- Initiate corrective measure +/− delayed IL-2, stop IL-2 if not easily reversible;
***Any absolute criteria***	- Initiate corrective measure +/− delayed IL-2, stop IL-2 if not easily reversible.

### Measurement of effect

#### Primary endpoint/translational endpoint

##### Immunological efficacy

Immunological efficacy will be assessed by quantifying circulating immune effectors that are specific for a selected panel of tumor antigens. This panel will include tumor antigens known to be expressed at high levels by over 80% of patients with melanoma or RCC and whose expression will confirmed in tumor biopsies taken before and after at least 2 cycles. Peptide libraries fully covering the sequences of tyrosinase, gp100 and Mart-1 for melanoma; 5 T4, CAIX/G250 and EGF-R for RCC; and survivin, MAGE-A3 and NY-ESO1 for both melanoma and RCC, will be utilized to stimulate peripheral blood mononuclear cells (PBMC) before and after therapy in IFN-g ELISPOT assays, according to the standard operating procedures currently used the our Somatic Cell Therapy Laboratory.

### Secondary endpoints

#### Predictive value of pretreatment serum biomarkers

Pretreatment concentrations of VEGF, fibronectin and of other relevant proinflammatory cytokines will be evaluated by SearchLight multiplex array analysis according to the manufacturer’s instructions (FDA-validated test). Serum sampling will be performed before each cycle to identify whether changes in marker values found during treatment can predict early therapy failure. The expression of CAIX in tumor tissue will be evaluated in pretreatment biopsies from RCC patients. In particular, both percentage and staining intensity will be recorded for each patient. In melanoma patients, tumor tissue removed before the start of treatment will be analyzed for the presence of N-Ras mutations.

#### Tumor assessment

This study will use ***immune-related response criteria (irRC)*** [45], a further refinement of mWHO criteria, to better document tumor response in subjects undergoing immunotherapy (Table 3). irRC were created because the natural history of clinical response observed in subjects treated with immunological agents, such as ipilimumab, differs from that observed in subjects receiving other classes of anti-cancer agents. The main differences between irRC criteria and traditional response criteria are as follows:

**Table 3:** **Immune-related response criteria (irRC)**

**Index lesions**	**Non-index lesions**	**New measurable lesions**	**New non -measurable lesions**	**% Change in tumor burden**	**Overall irRC response**
CR	CR	No	No	−100%	irCR
PR	Any	Any	Any	≥ −50%	irPR
PR	Any	Any	Any	< −50% to < +25%	irSD
PR	Any	Any	Any	≥ +25%	irPD
SD	Any	Any	Any	< −50% to < +25%	irSD
SD	Any	Any	Any	≥ +25%	irPD
PD	Any	Any	Any	≥ +25%	irPD
SD	Any	Any	Any	< −50% to < +25%	irSD
SD	Any	Any	Any	≥ +25%	irPD
PD	Any	Any	Any	≥ +25%	irPD

*irRC*: Immune-related response criteria; *CR*: Complete response; *PR*: Partial response; *SD*: Stable disease; *PD*: Progressive disease.

- measurable new lesions are incorporated into the tumor burden (*e.g.* added to the index lesions) and do not define progression unless the total measurable tumor burden increases by the required amount (25%).
- New non-measurable lesions (including bone lesions) are not considered progression if the total measurable tumor burden is stable or shrinking.
- Changes in non-measurable lesions contribute only in the definition of irCR.
- Progression of disease should be confirmed at two consecutive timepoints.

#### Radiologic assessment of tumor lesions

Contrast-enhanced CT scans of the brain, neck, chest, abdomen, pelvis and soft tissue (or MRI, if iodine contrast is medically contraindicated, *e.g.* for previous allergic reactions) will be performed for all patients during the Screening phase and at the time of tumor re-staging during the other study phases. CT scans of anatomic regions other than the chest, abdomen and pelvis must be performed in subjects in whom there is clinical suspicion of deep soft tissue metastases, *e.g.* lesions in the thigh.

Objective response or progression must be documented by the same imaging technique (CT scan or MRI) used during the Screening phase. The presence of progressive disease is not determined on the basis of only on a new lesion(s) found on bone scans. However, if bone lesions are identified at any time during the study, additional imaging studies of the lesion(s) must be performed to confirm the malignant nature of the new findings. Complete response must be confirmed by imaging studies showing the resolution of all metastatic lesions. Any subject who develops an objective tumor response (irCR or irPR) is required to undergo confirmatory scans no less than 4 weeks after the prior scan in order to verify the reliability of the radiologic finding.

#### Method and timing

Response will be calculated according to irRC criteria. For the purposes of this study, after baseline tumor staging performed on the day before the first treatment cycle, patients will be re-evaluated for response every 8 weeks during therapy and every 12 weeks during follow up. Confirmatory scans will also be performed no less than 4 weeks following initial documentation of objective response and before discontinuation of treatment due to irPD.

### Secondary clinical efficacy endpoints based on irRC

- ***Immune-related major durable disease control rate (irMDDCR)***: the proportion of treated subjects showing a disease control of 24 weeks measured from week 12, or from the date of the first overall response of irCR or irPR, until the date of irPD or death (whichever occurs first). For a subject who undergoes tumor resection following disease control but prior to disease progression, duration of disease control will be censored at the date of the last evaluable tumor assessment on or prior to the date of resection. Any subject who is unevaluable for MDDC for various reasons, *e.g.* censoring, lost to follow up, not assessable or unknown tumor assessments, will be considered a non-responder.
- ***Immune-related objective response rate (irORR)***: the proportion of treated subjects with an immune-related best overall response (irBOR) of confirmed irCR or confirmed irPR.
- ***Immune-related time to response (irTTR)***: the time from first dosing date until the measurement criteria (using irRC) are first met for overall response of irPR or irCR (whichever status comes first, and provided it is subsequently confirmed).
- ***Immune-related duration of response (irDOR)***: the time between the date on which the measurement criteria (using irRC) are first met for an irCR or irPR (whichever status comes first and provided it is subsequently confirmed) and the date of irPD or death (whichever comes first). For a subject who undergoes tumor resection following response but prior to disease progression, irDOR will be censored on the date of the last evaluable TA or prior to the date of resection.
- For subjects who are still alive and have no progressive disease, as assessed by the investigator using irRC, irDOR will be censored on the date of the last evaluable tumor assessment.
- ***Immune-related progression-free survival (irPFS)***: the time between the first dosing date and the date of irPD, or date of death, whichever occurs first (*i.e.* subjects who die without reported irPD will be considered to have progressed on the date of death). For subjects with no reported post-baseline tumor assessment, irPFS will be censored on the day of first dosing. For a subject who undergoes tumor resection following disease control but prior to disease progression, irPFS will be censored on the date of the last evaluable tumor assessment or prior to the date of resection. For subjects who are still alive and have no irPD, irPFS will be censored on the date of the last evaluable tumor assessment.
- ***Overall survival (OS)***: the time from randomization until the date of death. For those subjects who are still alive, OS will be censored at the recorded last date of subject contact, and for subjects with a missing recorded last date of contact, OS will be censored at the last date the subject was known to be alive.
- ***Immune-related time to progression (irTTP)***: the time from randomization to the first date of documented irPD (or death). Subjects without progression will be censored at their last tumor assessment date. Subjects without progression but who receive additional follow-up anticancer therapy will be censored on the date of their last tumor assessment prior to receiving the new therapy. Subjects with progression following additional anticancer therapy will also be censored on the date of their last assessment prior to receiving the therapy.
- ***Death on study***: any death occurring between the date of randomisation and up to 30 days after the end of treatment must be reported to the Coordinating Center within 24 hours as a serious adverse event (SAE), regardless of the relation to study drug(s). Deaths occurring during the study follow-up period (*i.e.* more than 30 days after the last vaccine dose) need only to be reported as an SAE if it is thought that there is a possible correlation with the study treatment(s). All deaths must be reported on the Death Report section of the CRF regardless of cause.

### Statistical considerations

#### Study design/endpoints

Single center, open-label proof-of-principle phase II trial to assess immune response and biomarkers potentially predictive of response. The primary objective of this study will be ***immunological efficacy***, as assessed by quantification of circulating immune effectors specific for a selected panel of tumor antigens. Secondary endpoints will be the predictive value of pretreatment serum biomarkers in identifying patients who are likely to benefit from high-dose IL-2-based therapy, toxicity, response rate (RR) and overall survival (OS). The analysis will be performed on an intention-to-treat population, *i.e.* all patients who have received at least one cycle of therapy. All time-dependent clinical endpoints (RR and OS) will be estimated with the Kaplan-Meier method. Frequency tables will be performed for all categorical variables. Continuous variables will be presented using mean and standard deviation or median and range.

Given the explorative intent of the study and the limited sample size, we are aware of being exposed to a high level of false-positive results. Unless otherwise indicated, analysis of demography and baseline characteristics will be performed on all enrolled patients. Demographic and laboratory results will be summarized using descriptive statistics. The percentage of patients reporting an adverse event (AE) up to 30 days after the end of HD-IL-2 treatment will be calculated with 95% confidence intervals by type of AE. The overall rate of grade 3–4 related AEs will be calculated. In addition, summary statistics of clinically significant laboratory abnormalities will be tabulated. The time of disease progression and the initiation of new therapies will also be documented.

### Sample size/accrual rate

This is a proof-of-principle trial with a minimax two-stage design:

- Step 1: 7 patients enrolled. A 40% immune response will preclude further study, whereas a 70% response rate will indicate that further study is warranted. Using alfa and beta errors of 0.10, if an immune response is observed in at least 3 of the 7 patients enrolled during the first stage, the study will go on with:
- Step 2: recruitment of 12 additional patients.

Treatments will be considered active if an immune response is observed in 11 out of 19 patients treated. An enrolment period of 20 months is planned, with an accrual rate of about 1 patient per month. There are no patient stratification factors planned.

### Study approval

The protocol, informed consent and any accompanying material provided to the patient were submitted by the investigator to the Local Ethics Committee for review. Approval from the Committee was obtained before starting the study (EudraCT no. 2012-001786- 32).

## Discussion

The development of target agents to treat melanoma and renal cell carcinoma has partially changed the history of patients with advanced disease. VEGF axis inhibitors have almost doubled the survival (from 12 to 22 months) of advanced RCC patients [46], as have BRAF inhibitors for advanced melanoma patients in a second-line setting [47]. However, these targeted agents rarely induce a complete and durable response. In contrast, HD-IL-2 has been shown to elicit durable responses ranging from 10% to 15% in melanoma patients and from 15% to 25% in those with RCC. Standard HD-IL-2 administration requires hospitalization and a dedicated team to monitor for capillary leak syndrome. Although patients often usually develop a flu-like syndrome and severe fatigue, these side-effects resolve after suspending the infusion, without sequelae. Toxicity from TKI or mTOR inhibitors for RCC and from chemotherapy, BRAF inhibitors or anti-CTLA-4 antibodies for melanoma are low-grade but continuous and exert a negative impact on quality of life. Furthermore, some toxicities have an initially mild clinical presentation and can, if not recognized, rapidly become severe and potentially life-threatening events [46,48-50]. If HD-IL-2 is to remain a component of a curative treatment strategy in patients with metastatic melanoma or RCC [51], its therapeutic index must be improved. Two current areas of research into immunotherapy are the elimination of immune suppression and the improvement of patient selection. Based on our understanding of mechanisms of action, some biomarkers of response to IL-2 could help to identify a subset of patients who would probably benefit from treatment.

Up to now, the majority of putative predictors of IL-2 response have been post-treatment variables, such as the height of rebound lymphocytosis, treatment induced thrombocytopenia, the development of autoimmune thyroiditis and vitiligo, and the decrease in the absolute number and frequency of peripheral Tregs. In RCC, the level of carboxic anhydrase IX in primary tumors has been found to be a potentially useful pretreatment predictor. In both melanoma and RCC, (high levels of) serum vascular endothelium growth factor (VEGF) and fibronectin have also been identified as independent predictors of non-response and poorer overall survival. Other analyses of pretreatment factors have correlated IL-2 with the Cw7 phenotype and low serum levels of IL-6 and C-reactive protein, but none have proven to be sufficiently predictive of clinical benefit to be useful in selecting candidates for IL-2 therapy [43,44].

## Conclusions

As long as HD-IL-2 remains a necessary component of a curative treatment strategy in patients with metastatic melanoma or RCC, we must strive to improve its therapeutic index. Two current areas of investigation into immunotherapy focus on the elimination of immune suppression/regulation and the improvement in patient selection. Based on our understanding of the mechanisms of action, biomarkers of response to IL-2 might allow us to accurately select a subset of patients who will benefit from treatment. The main aims of this study are to improve patient selection and reduce toxicity, whilst also guaranteeing the same clinical benefit. In an attempt to develop a more manageable and better tolerated therapeutic schedule it has been decided to try a short IL-2 infusion repeated every 21 days, to add corticosteroids [38] to reduce the risk of capillary leak syndrome, and to add radiotherapy to exploit the abscopal effect [52,53].
